# LncRNA SPRY4‐IT1 facilitates cell proliferation and angiogenesis of glioma via the miR‐101‐3p/EZH2/VEGFA signaling axis

**DOI:** 10.1002/cam4.5517

**Published:** 2022-12-07

**Authors:** Ji Wang, Yanming Chen, Qing Wang, Hui Xu, Qianqian Jiang, Man Wang, Shenggang Li, Ying Chen, Chunwang Wu, Pei Yu, Zongyu Xiao, Wenjin Chen, Qing Lan

**Affiliations:** ^1^ Department of Neurosurgery The Second Affiliated Hospital of Soochow University Suzhou China; ^2^ Department of Neurosurgery Dushu Lake Hospital Affiliated to Soochow University Suzhou China; ^3^ Department of Neurosurgery Peking University Shenzhen Hospital Shenzhen China

**Keywords:** angiogenesis, EZH2, glioma, miR‐101‐3p, proliferation, SPRY4‐IT1, VEGFA

## Abstract

**Background:**

SPRY4‐IT1 (SPRY4 intronic transcript 1) is a long non‐coding RNA (lncRNA) that has been identified as a novel oncogene in various cancers, including glioma. However, its function and underlying mechanism in glioma remain largely unclear. Here, we investigated the role of SPRY4‐IT1 in the development of glioma and its underlying mechanism.

**Methods:**

Bioinformatics analysis and RT‐qPCR assay were used to examine the expression of SPRY4‐IT1 in glioma tissues. The CCK‐8, EdU, and Xenograft tumor assays wereperformed to assess the proliferation effect of glioma cells. The tube forming assay and Chick Embryo Chorioallantoic Membrane (CAM) assay were conducted to detect the angiogenesis effect of HUVECs. RNA‐sequencing, western blotting, RT‐qPCR, ELISA, and IHC assays were employed to verify the regulatory mechanism of the SPRY4‐IT1/ miR‐101‐3p/EZH2/VEGFA axis.

**Results:**

Analysis of the TCGA dataset and data from our own cohort demonstrated that SPRY4‐IT1 was overexpressed in patients with glioma, and high SPRY4‐IT1 expression correlated with poor prognosis. In vitro and in vivo experiments showed that SPRY4‐IT1 promoted the proliferation of glioma cells. RNA sequencing and Gene Ontology (GO) enrichment analysis indicated significant enrichment of angiogenesis. HUVEC tube forming assay and CAM assay confirmed that SPRY4‐IT1 could induce angiogenesis of glioma cells in vitro and in vivo. Mechanistically, SPRY4‐IT1 upregulated EZH2 expression by sponging miR‐101‐3p to induce VEGFA expression in glioma cells. Moreover, SPRY4‐IT1 activated the VEGFR2/AKT/ERK1/2 pathway in HUVECs mediated by glioma cells. Rescue experiments further confirmed that SPRY4‐IT1 promoted glioma cell proliferation and angiogenesis via the miR‐101‐3p/EZH2/VEGFA signaling axis.

**Conclusions:**

Our findings provide compelling evidence showing that SPRY4‐IT1 upregulated EZH2 to induce VEGFA by sponging miR‐101‐3p, thereby achieving cell proliferation and angiogenesis in glioma. Therefore, targeting SPRY4‐IT1/miR‐101‐3p/EZH2/VEGFA axis may improve the outcomes of patients with glioma.

## Graphical abstract

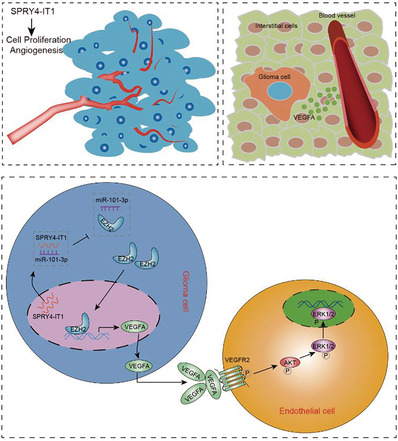

## 1 INTRODUCTION

Glioma, the most common primary malignant tumor of the central nervous system (CNS), represents nearly 25.13% of all primary CNS tumors. Glioblastoma (GBM) is the most aggressive type and most common form of glioma in adults, accounting for 57.7% of all gliomas.^1^ Despite current advances in multimodal therapy (surgery, radiotherapy, and chemotherapy) for GBM, GBM patients still suffer from poor prognoses (5‐year survival rate of only 5.8%).^2^ Therefore, alternative approaches to improve the treatment of glioma are urgently needed.

The vast majority of the human genome is transcribed. However, approximately 98% of transcripts do not encode for any proteins and are termed non‐coding RNAs (ncRNAs). Long non‐coding RNA (lncRNA) accounts for approximately 80%–90% of the ncRNAs, has transcripts longer than 200 nucleotides and was once regarded as junk DNA.^3^ However, lncRNA has been increasingly recognized to play large regulatory roles in the biological processes.^4^ To date, dysregulation of lncRNA has been linked to every cancer investigated and affects all major cancer hallmarks.^5^, ^6^ Additionally, increasing evidence has indicated that lncRNA plays a critical role in the tumorigenesis, progression, and drug resistance of glioma.^7^ SPRY4‐IT1 (SPRY4 intronic transcript 1), derived from an intron region within the SPRY4 gene, which was confirmed to be localized in the cytoplasm of tumor cells, has attracted increasing attention among various lncRNA.^8^, ^9^, ^10^ SPRY4‐IT1 was first discovered as an oncogene modulating melanoma cell apoptosis and invasion.^8^ In recent years, genome‐wide analysis and molecular functional studies have focused on the role of SPRY4‐IT1 in cancer progression.^9^, ^11^, ^12^ Shojaei et al. found that SPRY4‐IT1 might be a novel oncogenic lncRNA by reviewing the biological function and mechanism of SPRY4‐IT1 in cancer progression.^13^ Increasing evidence has also suggested SPRY4‐IT1 as a prognostic factor for poor outcomes as it was associated with accelerating glioma cell proliferation, invasion, and migration via the epithelial‐mesenchymal transition.^14^, ^15^ However, the mechanism of SPRY4‐IT1 in glioma remains unclear.

In this current study, we assessed the prognostic value of SPRY4‐IT1 in patients with glioma and explored its underlying molecular mechanisms in glioma cells and animal models. We found that SPRY4‐IT1 was upregulated in glioma specimens and correlated with a poor prognosis in glioma patients. Moreover, SPRY4‐IT1 could promote glioma cell proliferation and glioma cell‐induced angiogenesis in vitro and in vivo. Further, we confirmed that SPRY4‐IT1 regulated the miR‐101‐3p/EZH2/VEGFA signaling axis to induce tumorigenesis and angiogenesis in glioma. Taken together, our findings provided insights into the role, prognostic significance and underlying mechanism of SPRY4‐IT1 in glioma.

## 2 MATERIALS AND METHODS

### 2.1 Clinical samples

Fifty‐nine samples of human glioma tissues (WHO II grade: 15 samples; WHO III grade: 21 samples; WHO IV grade: 23 samples) were used in this study. They were both pathologically diagnosed as gliomas for the first time from February 2017 to September 2020 at the Department of Neurosurgery, the Second Affiliated Hospital of Soochow University (Suzhou, Jiangsu, China). They did not receive any chemotherapy or radiotherapy before surgery. Their clinicopathological features are shown in Table S1. Another dataset containing three samples from brain trauma patients was collected as the control group. All patients/participants provided written informed consent to participate in this study.

### 2.2 Cell culture

Human glioma cell lines (U87, SNB19, and U118) and normal human astrocytes (NHA) were provided by Dr. Li Ming (Department of Neurosurgery, University of Minnesota) and cultured in Dulbecco's Modified Eagle's Medium (DMEM) containing 10% fetal bovine serum (FBS). Human umbilical vein endothelial cells (HUVECs) were obtained from Procell Life Science Company and maintained in a DMEM/F12 (1:1) medium containing 10% FBS. All cells were cultured at 37°C in a humid atmosphere with 5% CO_2_.

### 2.3 Cell transfection

Two siRNAs targeting SPRY4‐IT1 (#1 5′‐GCTTTCTGATTCCAAGGCCTATTAA‐3′, #2 5′‐GGTGGTTGAAAGGAATCCT‐3′); two siRNAs targeting EZH2 (#1 5′‐GGAUGGUACUUUCAUUGAAGA‐3′, #2 5′‐GCAAAGUACUGUAAGAAUAAU‐3′); and a miR‐101‐3p inhibitor (5′‐UUCAGUUAUCACAGUACUGUA‐3′) were synthesized by GenePharma. Plasmids overexpressing SPRY4‐IT1 and EZH2 were constructed based on the pcDNA3.1 vector, purchased from the GeneCreate Biological Company. The glioma cells were seeded in six‐well plates and transfected when the cells reached a confluence of 60%–70% on the second day. Cell transfection was carried out using Lipofectamine 3000 (Invitrogen) according to the manufacturer's protocol. The cells were collected for subsequent experiments 48 h post‐transfection.

### 2.4 Lentivirus packaging and generation of stable cells

The short hairpin RNA of SPRY4‐IT1 (shSPRY4‐IT1) (CCGGGGTGGTTGAAAGGAATCCTCTCGAGAGGATTCCTTTCAACCACCTTTTTG) was inserted into the hU6‐MCS‐Ubiquitin‐IRES‐puro plasmid. Lentivirus packaging was performed according to a previously described method.^16^ HEK 293T cells were co‐transfected with a shSPRY4‐IT1 plasmid, Pcmvdr8.91 plasmid, and pMD.G‐VSV‐G plasmid. The cell supernatants were harvested and filtered with a 0.45 μm nitrocellulose filter 48 h post‐transfection. Finally, the glioma cells were infected with the supernatants for 48 h and screened with 5 μg/mL puromycin for 1–2 weeks to obtain stable cells.

### 2.5 Collection of the glioma conditioned medium (GCM)

The collection of GCM was performed as previously mentioned.^17^ The glioma cells were grown to 80% confluence under different treatments, and the culture medium was changed with fresh serum‐free DMEM for 24 h. Then, the supernatants were harvested, centrifuged at 2000 *g* for 10 min at 4°C to remove cellular debris, and supplemented with 0.5% fresh serum. The GCM was immediately used for subsequent experiments.

### 2.6 Cell counting kit‐8 (CCK8) assay

Cell viability was assessed using a CCK8 kit. The cells were trypsinized, counted, and seeded into 96‐well plates (2 × 10^3^ cells/well) 24 h post‐transfection. At pre‐designed time points (0, 24, 48, 72, 96, and 120 h), the cells were incubated with DMEM/10% FBS containing 10% CCK‐8 solution (Dojindo) at 37°C for 1 h. The absorbance of cells was measured at 450 nm using a microplate reader (Infinite 200 PRO NanoQuant, TECAN).

### 2.7 EdU assay

Cell proliferation was assessed using an EdU kit (RiboBio, Guangzhou, China) following the manufacturer's protocol. The glioma cells were incubated with EdU solution for 4 h, then dyed with Apollo Dye solution after being fixed with 4% paraformaldehyde (PFA). Photographs of EdU cells were captured with a confocal microscope (Zeiss), and the EdU‐positive cell rate was quantified using the ImageJ software (NIH).

### 2.8 Transwell assay

The transwell assay was performed to measure cell migration ability. The cells (2 × 10^4^/well in 200 μl DMEM) were plated in the upper chamber, and GCM (600 μl) was added to the lower chamber to stimulate cell migration. After 24 h, the remaining cells in the upper chamber were removed with a cotton swab, while the cells in the lower chamber were fixed with 4% PFA and stained with 0.1% crystal violet methanol. The number of migrated cells was quantified by counting the cells in six random microscopic fields (×100 magnification).

### 2.9 Wound healing assay

HUVECs were seeded in six‐well plates and allowed to reach a 90–100% confluence on the second day. Wounds were created with a 1.0‐ml pipette tip by scratching straight lines on the cell surface, and detached cells were removed by washing with FBS‐free DMEM. HUVECs were incubated with GCM for 48 h, wound images were captured under a light microscope (EVOS), and the wound area was analyzed using the ImageJ software.

### 2.10 Tube forming assay

A precooled 24‐well plate was coated with 50% Matrigel (100 μl /well) (Corning) and was left to polymerize at 37°C for 30 min. HUVECs (1 × 10^5^/well) were suspended in GCM supplemented with 10% FBS and seeded in 24‐well plates for 12 h. Then, tube structures were photographed by a light microscope. Tubules were quantified and analyzed using the ImageJ software.

### 2.11 Chick embryo chorioallantoic membrane (CAM) assay

The CAM assay was used to investigate angiogenesis effects following a previously described protocol.^18^ Briefly, after incubation at 37°C for 8 days, a 1.0‐cm window was created on the air sac of an egg (Linrui Breeding Co. Ltd) to expose the chorioallantoic membrane. A 0.5‐cm diameter filter paper was first placed on top of the CAM, and 100 μl of GCM was added to the center of the paper. The window was covered with adhesive tape, and the eggs were incubated in a humid incubator at 37°C for 48 h. On day 10, blood vessels on the CAM were photographed with a digital camera (Canon). The numbers of second‐ and third‐order vessels within the defined area of the membrane were manually counted to assess the effects of GCM on angiogenesis.

### 2.12 Enzyme‐linked immunosorbent assay (ELISA)

The concentration of VEGFA in the glioma cells medium was determined using an ELISA kit (Elabscience) according to the manufacturer's protocol. The absorbance was measured using a microplate reader at 450 nm.

### 2.13 RNA isolation and quantitative real‐time PCR assay

Total RNA was isolated from the cells and tissues by a TRIZOL kit (Invitrogen) based on the manufacturer's protocol. RNA was quantified and then reverse‐transcribed into cDNA by a cDNA synthesis kit (Novoprotein). qRT‐PCR was conducted by SYBR Green (Novoprotein) in an ABI 7000 thermal cycler. RNA expression was analyzed using the 2^−ΔΔCT^ method. GAPDH or U6 was used as the endogenous control for normalization. The primer sequences were synthesized by Tsingke Biotechnology. The sequence of the primers is outlined in Table S2.

### 2.14 Western blot assay

The glioma cells were lysed using RIPA buffer with Protease and Phosphatase inhibitor (Beyotime). The total protein concentration was examined using a BCA kit. Equal amounts of total protein (30 μg) were electrophoresed in 10%–12% SDS‐PAGE and electro‐transferred onto NC membrane (GE). After blocking with 5% non‐fat dry milk in TBST for 1 h at room temperature (RT), the membranes were incubated with primary antibodies at 4°C overnight. Then, the membranes were incubated with HRP‐labeled secondary antibodies (Proteintech) for 1 h at RT. An enhanced chemiluminescence reagent (Millipore) was used to detect protein expression value. The relative quantity of proteins was analyzed using the Image J software. The primary antibodies used are as follows: anti‐EZH2 (1:2000, #66476‐1‐Ig, Proteintech), anti‐VEGFA (1:1000, #66828‐1‐Ig, Proteintech), anti‐VEGFR2 (1:200, #sc‐6251, Santa‐Cruz Technology), anti‐Phospho^Tyr1175^‐VEGFR2 (1:1000, #2478, CST), anti‐AKT (1:1000, #9272, CST), anti‐ Phospho^Thr308^‐AKT (1:1000, #13038, CST), anti‐ERK1/2 (1:1000, #4695, CST), anti‐Phospho^Thr202/Tyr204^‐ERK1/2 (1:1000, #4370, CST), anti‐GAPDH (1:5000, #60004‐1‐Ig, Proteintech), and anti‐β‐tubulin (1:5000, #10094‐1‐AP, Proteintech).

### 2.15 RNA sequencing

Total RNA was isolated using an RNeasy kit (Qiagen) from three independent replicates of the U87 cells with SPRY4‐IT1 knockdown or control. The RNA amount and purity of each sample were quantified. Further RNA sequencing and result analysis were executed by LC‐Bio Technology Co., Ltd.

### 2.16 Immunohistochemistry (IHC) and hematoxylin–eosin (HE) staining

Immunohistochemical staining was performed using an immunohistochemistry kit (#PK‐4002, ZSGB‐BIO). Briefly, xenograft tumor mouse brains were fixed in 4% PFA and embedded in paraffin. Then, 5 μm‐thick slices were cut with a microtome (Leica), then deparaffinized, dehydrated, and incubated in heat‐mediated antigen retrieval. Subsequently, the endogenous catalase was eliminated with 3% H2O2‐methanol, and tissue slices were incubated with indicated primary antibodies against EZH2 (1:200, #ab191080, Abcam), VEGFA (1:400, #66828‐1‐Ig, Proteintech), and CD31 (1:2000, #ab182981, Abcam) at 4°C overnight. After washing with PBS, the sections were incubated with biotinylated secondary antibodies for 1 h at RT. They were then incubated with peroxidase solution for 30 min before the sections were colored with the DAB reagent and counterstained with hematoxylin. Images of each section were captured and analyzed with a light microscope at ×100 magnification.

HE staining was performed with an HE kit (BaSO Biotechnology). The paraffin‐embedded sections were sequentially deparaffinized, dehydrated, stained by hematoxylin, differentiated by the addition of hydrochloric ethanol, backed to blue with ammonia water, and stained with eosin. Afterward, the sections were dehydrated, cleared, and sealed. Finally, tissue HE photographs were acquired under a light microscope.

### 2.17 Xenograft tumor assay

U87 shSPRY4‐IT1 cells or scramble cells (3 × 10^5^ in 10 μl PBS) were injected intracranially into female 6‐week‐old BALB/c nude mice (5 mice for each group). The mice were observed daily for death or neurological symptoms and were subsequently sacrificed when they exhibited neurological symptoms. Then, the whole brain was harvested, paraformaldehyde‐fixed, paraffin‐embedded, and sectioned coronally from anterior to posterior. The section with the largest tumor cross‐sectional area was selected for tumor size measurement. The tumor volume was calculated using the formula: *V* = (*a* × *b* ^2^)/2, where *a* and *b* represent the longest diameter and shortest diameter, respectively. Parameters *a* and *b* were measured using the Image J software.

### 2.18 Bioinformatics analysis

Glioma TCGA datasets including SPRY4‐IT1 expression values and patient survival were downloaded from the GlioVis website (https://gliovis.bioinfo.cnio.es/). The expression of SPRY4‐IT1 and clinical data of glioma samples (WHO II: 194 samples, WHO III: 218 samples, WHO IV: 161 samples) were extracted and used for the survival analysis. The samples were divided into high and low groups according to the median SPRY4‐IT1 expression value.

### 2.19 Statistical analysis

Statistical analyses were conducted using GraphPad Prism 8.0. Bars and error represent the mean ± standard deviation (mean ± SD) of replicate measurements. The means of normally distributed continuous data between two groups were analyzed using unpaired Student's t‐tests. One‐way ANOVA was used to compare SPRY4‐IT1 expression levels between different clinical grades of glioma. The Chi‐square (*χ* ^2^) test was used to analyze the association between SPRY4‐IT1 expression and clinicopathological characteristics of glioma. Survival curves were plotted using the Kaplan–Meier method and compared using the log‐rank test. Statistical significance was defined as *p* < 0.05.

## 3 RESULTS

### 3.1 LncRNA SPRY4‐IT1 expression is elevated in glioma and correlated with poor patient prognosis

**FIGURE 1:**
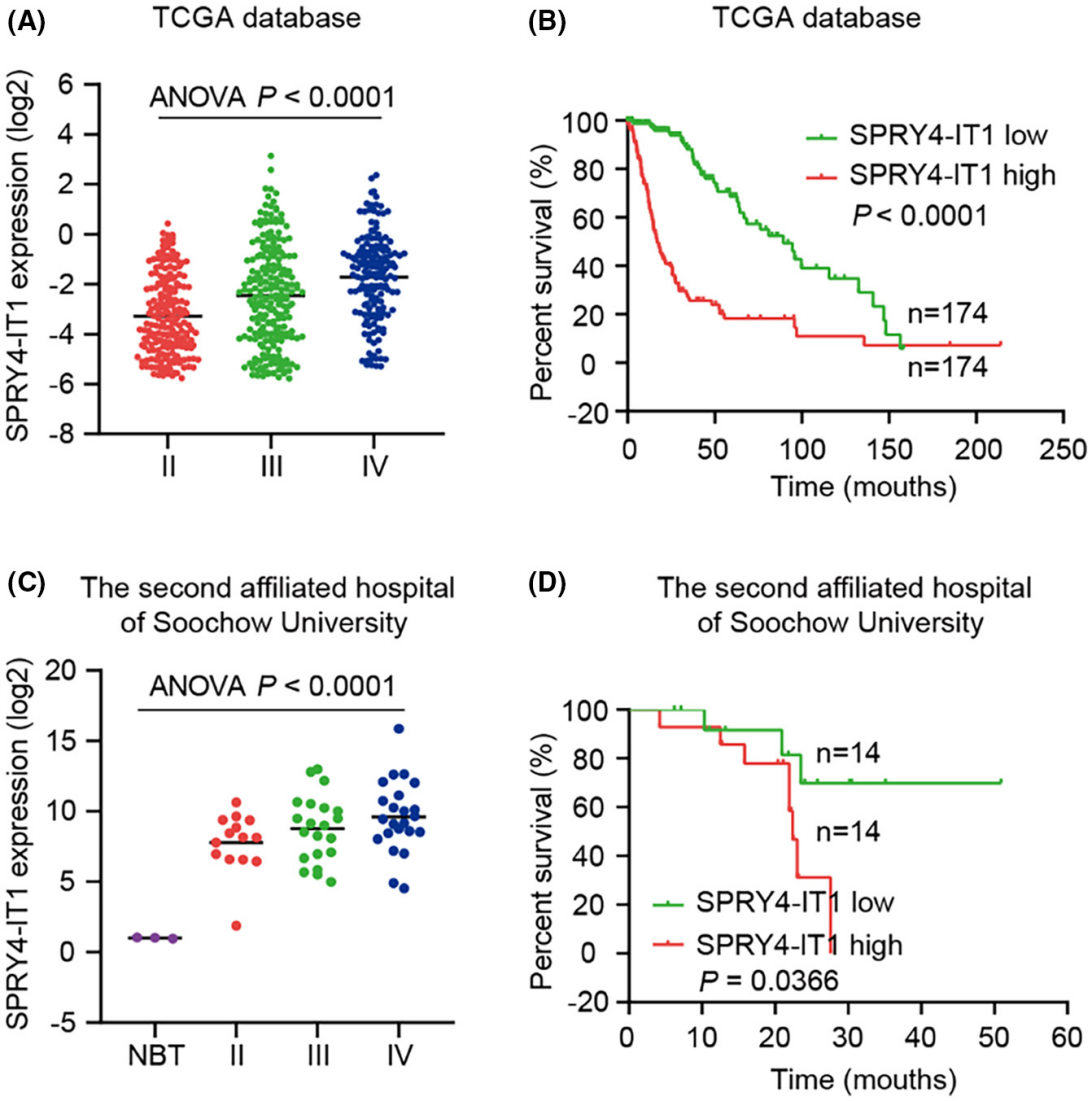
LncRNA SPRY4‐IT1 is elevated in the glioma tissue and predicts poor patient prognosis. (A) SPRY4‐IT1 expression in different grades of glioma specimens from the TCGA database. (B) The Kaplan–Meier plots of glioma patient prognosis based on SPRY4‐IT1 expression value from the TCGA database. (C) qRT‐PCR analysis of SPRY4‐IT1 levels in normal brain tissues and different pathological grades of gliomas from our own cohort. (D) Kaplan–Meier plots of glioma patients' overall survival data according to high versus low expression of SPRY4‐IT1 from our own cohort.

To understand the expression profiles of lncRNA SPRY4‐IT1 in glioma tissues and their prognostic significance, we first analyzed the TCGA sequencing data of glioma. The data demonstrated that SPRY4‐IT1 mRNA increased along with WHO grades and was associated with poor outcomes in glioma patients (Figure 1A,B). Subsequently, qRT‐PCR was performed to detect SPRY4‐IT1 expression in fresh glioma specimens with different WHO grades and non‐brain tumors. The data revealed that SPRY4‐IT1 expression was dramatically elevated in glioma tissues and gradually increased with WHO grades (Figure 1C). In addition, we carried out a survival statistic (Kaplan–Meier analysis) to assess the relation between SPRY4‐IT1 expression and survival time based on glioma patients' follow‐up information. We found that glioma patients with higher SPRY4‐IT1 levels had a shorter survival time (Figure 1D). Further, according to the medium cutoff value of SPRY4‐IT1 expression in glioma tissues, the patients were split into high and low expression groups. The glioma patients with a higher tumor grade exhibited higher SPRY4‐IT1 expression (Table S1). Thus, these data suggest that SPRY4‐IT1 overexpression could independently indicate poor prognosis in glioma patients.

### 3.2 LncRNA SPRY4‐IT1 promotes glioma cell proliferation in vitro

**FIGURE 2:**
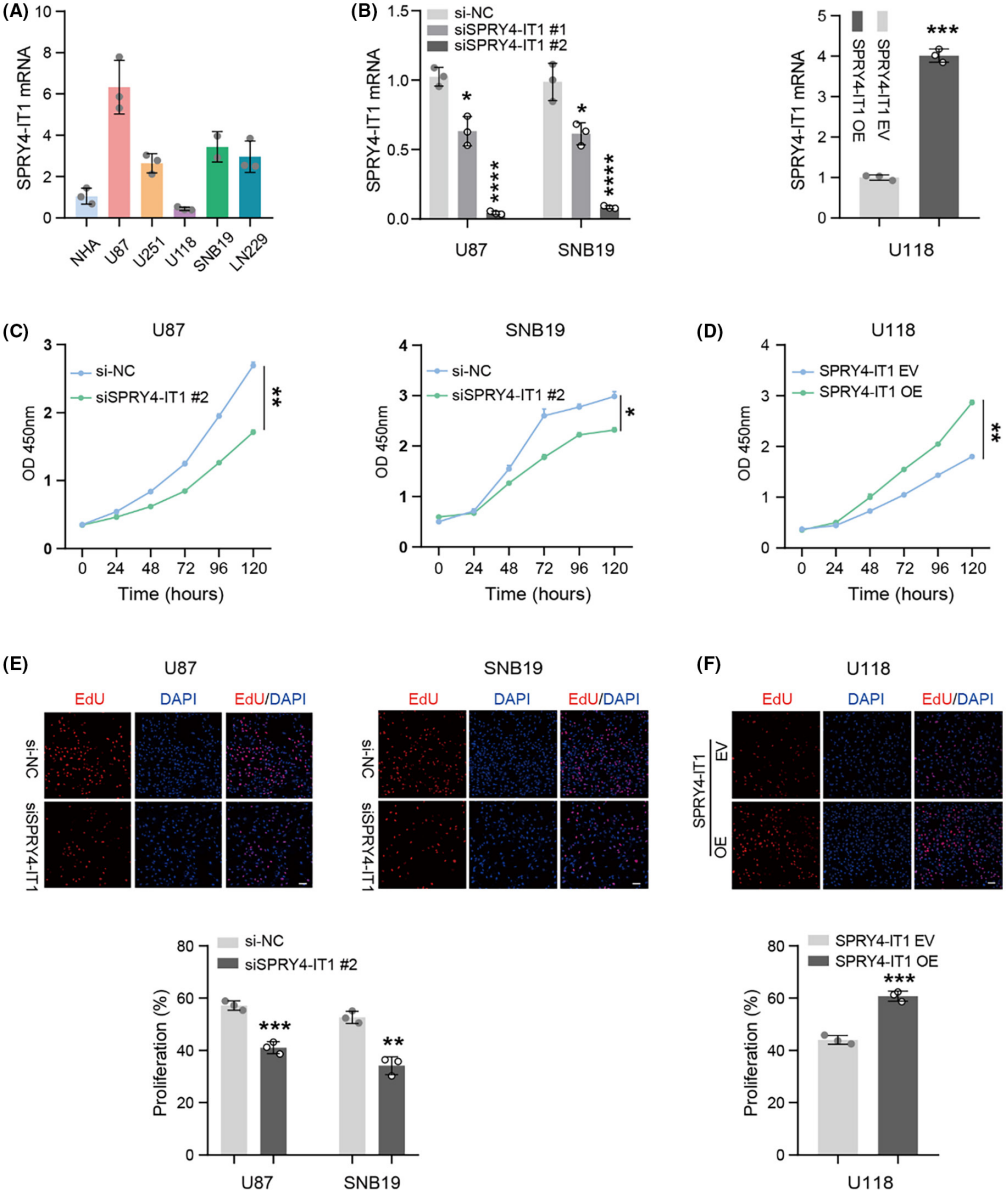
LncRNA SPRY4‐IT1 induces glioma cell proliferation in vitro. (A) qRT‐PCR analysis of SPRY4‐IT1 levels in glioma cell lines (U87, U251, U118, SNB19, and LN229) and NHA. (B) SPRY4‐IT1 silencing and overexpression efficiency were determined using qRT‐PCR analysis in glioma cells (U87 and SNB19; U118). (C, D) CCK‐8 assays were conducted to examine cell proliferation in SPRY4‐IT1‐silenced cells (U87 and SNB19) and SPRY4‐IT1‐overexpressed cells (U118). (E, F) EdU assays were conducted to detect cell proliferation rate in SPRY4‐IT1‐silenced cells (U87 and SNB19) and SPRY4‐IT1‐overexpressed cells (U118). Scale bar: 50 μm. *p < 0.05, **p < 0.01, ***p < 0.001 and ****p < 0.0001.

qRT‐PCR analysis of SPRY4‐IT1 mRNA levels in glioma cell lines and NHA showed that high expression values of SPRY4‐IT1 were evident in most glioma cell lines (U87, U251, SNB19, and LN229) compared with NHA (Figure 2A). It demonstrated that SPRY4‐IT1 was highly expressed in glioma cell lines, in line with the observation in fresh glioma specimens. To verify the molecular functions of lncRNA SPRY4‐IT1 in glioma cells, U87, SNB19, and U118 cells were chosen for subsequent experiments. First, we silenced SPRY4‐IT1 expression using siRNA transfection in U87 and SNB19 cells, or overexpressed SPRY4‐IT1 by pcDNA3.1 plasmid transfection in U118 cells. qRT‐PCR was used to analyze the efficiency of knockdown or overexpression (Figure 2B). Next, a CCK‐8 assay was conducted to evaluate cell proliferation viability. The data revealed that silencing of SPRY4‐IT1 suppressed U87 and SNB19 cells' growth, and overexpression of SPRY4‐IT1 enhanced U118 cells' proliferation (Figure 2C,D). Then, an EdU assay was conducted to further examine the effect of SPRY4‐IT1 on cell proliferation. Consistently, the data indicated more EdU‐positive cells when SPRY4‐IT1 was overexpressed, and the inhibition of SPRY4‐IT1 significantly decreased the number of EdU‐positive cells (Figure 2E,F). Collectively, these data indicate that SPRY4‐IT1 could induce glioma cell proliferation in vitro.

### 3.3 LncRNA SPRY4‐IT1 enhances the ability of glioma cells to induce angiogenesis

**FIGURE 3:**
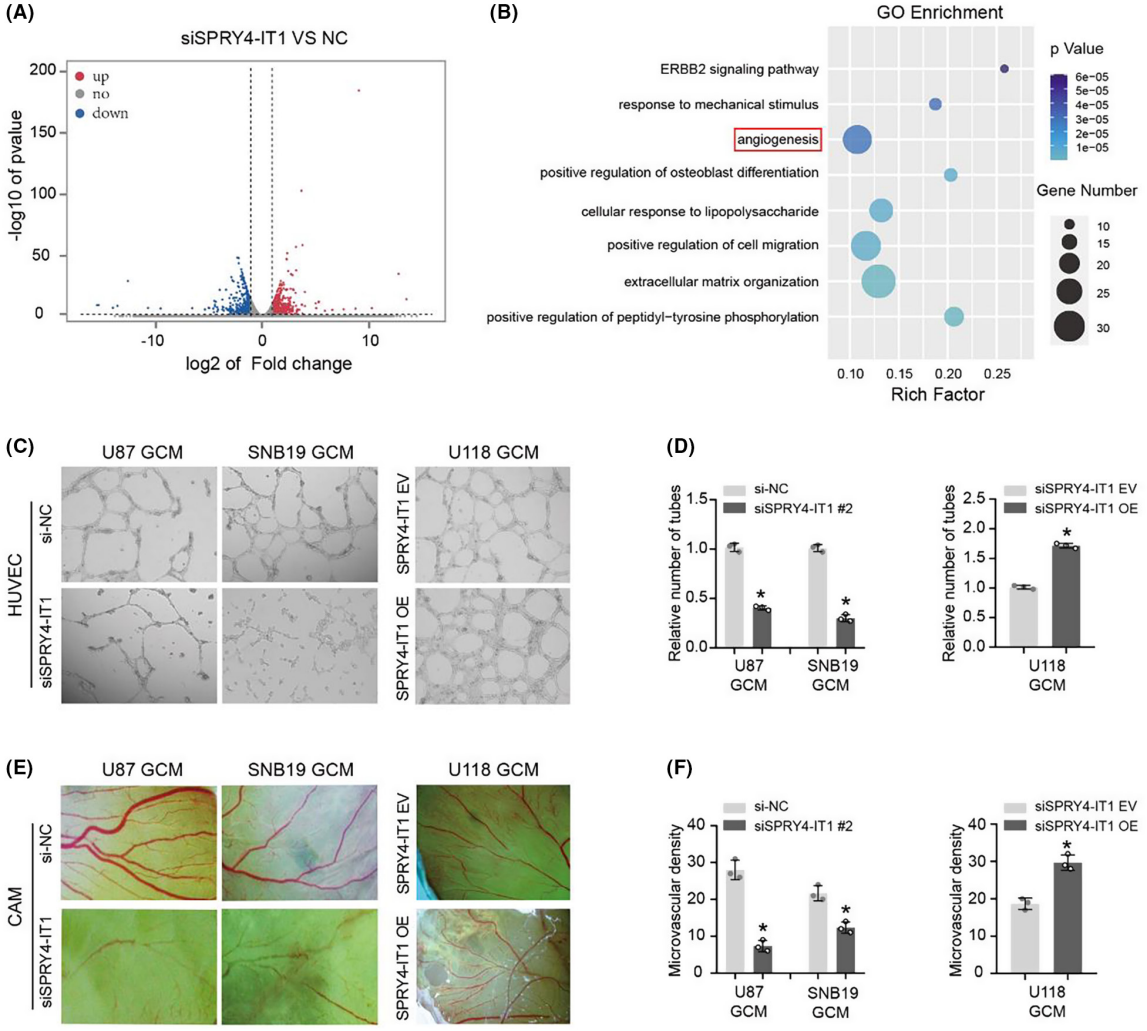
LncRNA SPRY4‐IT1 enhances the ability of glioma cells to induce angiogenesis. (A) Volcano plot of RNA‐sequencing demonstrating the upregulation of 739 genes and downregulation of 508 genes in U87 siSPRY4‐IT1 cells compared to the control cells (>2 folds, *p* < 0.05, one‐way ANOVA test). (B) Gene Ontology (GO) analysis showing significantly enriched terms based on differential gene expression. (C) Effects of SPRY4‐IT1 knockdown and overexpression GCM on tube formation of HUVECs measured by Matrigel tube formation assay in vitro. (D) Statistical analysis of HUVECs tube formation. (E) CAM assays were performed to investigate the effect of CM from glioma cells transfected with siSPRY4‐IT1 or NC and oe‐SPRY4‐IT1 or vector on angiogenesis in vivo. (F) Statistical analysis based on angiogenesis. *p < 0.05.

To further investigate the molecular mechanism of how SPRY4‐IT1 promoted glioma cell proliferation, RNA‐sequencing was performed to compare the transcriptomes of U87 siSPRY4‐IT1‐NC and U87 siSPRY4‐IT1 cells. The Volcano plot demonstrated that 739 genes were upregulated and 508 genes were downregulated, compared with U87 siSPRY4‐IT1‐NC cells (>2 folds, *p* < 0.05, one‐way ANOVA test) (Figure 3A). Based on these differentially expressed genes, GO enrichment analysis revealed that the extracellular matrix organization, cell migration regulation and, angiogenesis were significantly enriched (Figure 3B). Previous studies have confirmed that SPRY4‐IT1 could regulate cell proliferation and migration in various tumors,^19^, ^20^ including glioma.^15^ However, whether lncRNA SPRY4‐IT1 could regulate tumor angiogenesis is yet unknown. Thus, we performed glioma cell‐mediated vascular endothelial cell tubule formation assays. The results revealed that the culture medium (CM) from SPRY4‐IT1 silenced U87 and SNB19 cells significantly induced less tubule formation of HUVECs compared with the control group, while CM of SPRY4‐IT1 overexpressed U118 cells strongly stimulated more tubule formation of HUVECs than the control group (Figure 3C,D). Meanwhile, to investigate the effect of SPRY4‐IT1 on angiogenesis in vivo, we conducted a CAM assay. Treatment with CM from the SPRY4‐IT1‐silenced U87 and SNB19 cells decreased the vessel number compared with the control group, while CM from SPRY4‐IT1‐overexpressed U118 cells promoted the formation of more vessels than the control group (Figure 3E,F). Therefore, these results indicate that SPRY4‐IT1 enhanced glioma cell‐mediated angiogenesis.

### 3.4 LncRNA SPRY4‐IT1 promotes the ability of glioma cells to induce proliferation and migration of HUVECs

**FIGURE 4:**
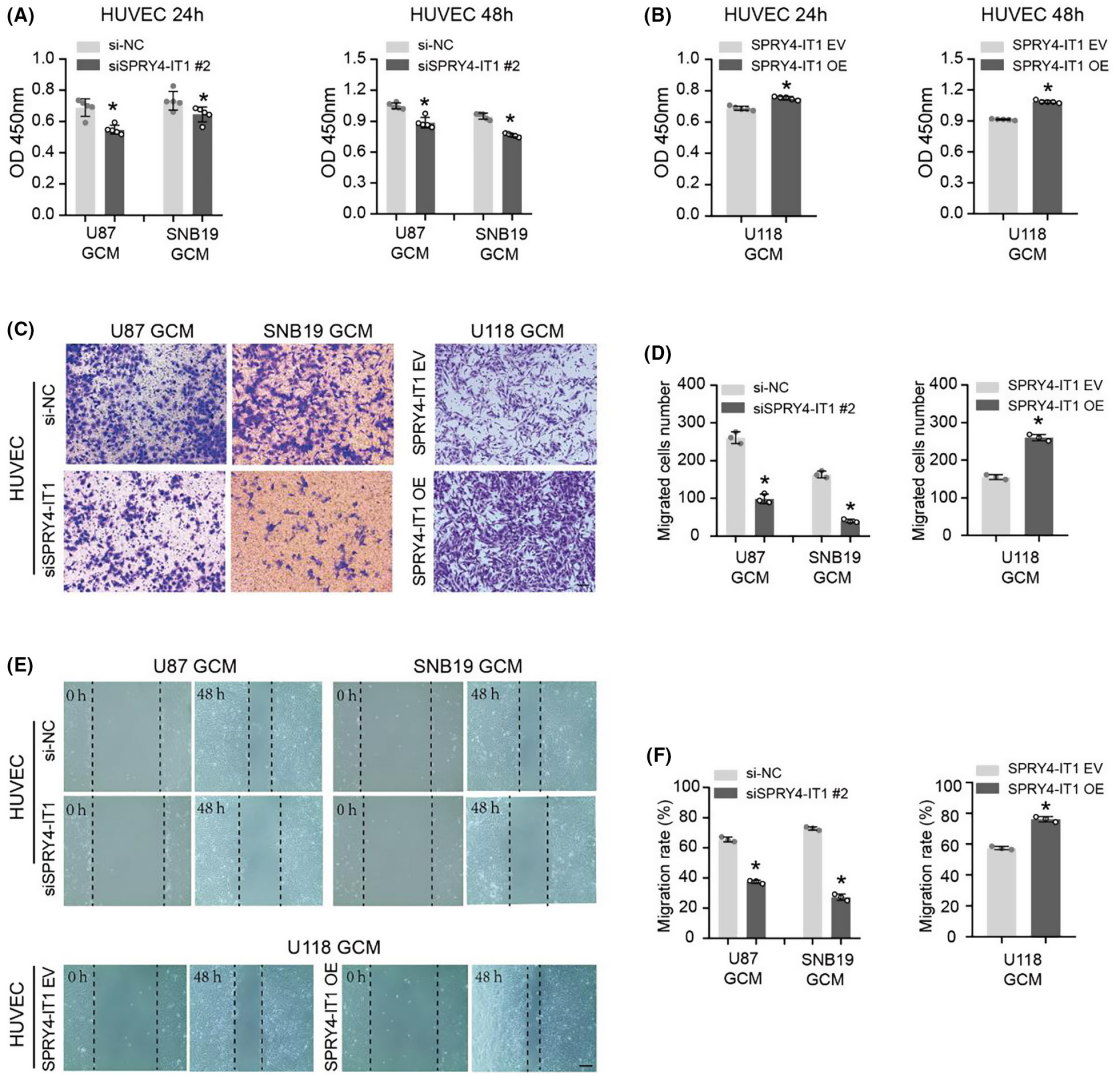
LncRNA SPRY4‐IT1 promotes the ability of glioma cells to induce the proliferation and migration of HUVECs. (A, B) CCK‐8 assays were conducted to determine the growth of HUVECs treated with SPRY4‐IT1‐knockdown and SPRY4‐IT1‐overexpressed GCM at 24 h and 48 h. (C) Effects of SPRY4‐IT1‐knockdown and SPRY4‐IT1‐overexpressed GCM on the migration of HUVECs were detected using transwell assays. Scale bar: 100 μm. (D) Quantitative analysis of migrated HUVECs number. (E) Wound‐healing assays were performed to assess the migration of HUVECs treated with SPRY4‐IT1‐knockdown and SPRY4‐IT1‐overexpressed GCM at 48 h. Scale bar: 200 μm. (F) Statistical analysis of HUVECs migration rate. *p < 0.05.

Here, we investigated the potential role of lncRNA SPRY4‐IT1 in endothelial cell proliferative and migrative functions, which are critical for angiogenesis. CM of SPRY4‐IT1 silenced U87 and SNB19 cells effectively suppressed the proliferation of HUVECs, while CM of SPRY4‐IT1‐overexpressed U118 cells promoted the proliferation of HUVECs (Figure 4A,B). Then, we evaluated the effect of CM on HUVECs migration by transwell assay, and the data revealed that CM derived from SPRY4‐IT1‐knockdown U87 and SNB19 cells could weaken the migration of HUVECs. In contrast, CM from SPRY4‐IT1‐overexpressed U118 cells stimulated the migration of HUVECs (Figure 4C,D). Consistent with the transwell assay, similar results were observed for the scratch wound assay. As depicted in Figure 4E, the results demonstrated that CM from SPRY4‐IT1‐knockdown glioma cells significantly decreased HUVECs migration distance, and CM from SPRY4‐IT1‐overexpressed glioma cells markedly increased HUVECs migration distance (Figure 4E,F). These findings demonstrated that SPRY4‐IT1 has a key role in inducing endothelial cell proliferation and migration in glioma cells.

### 3.5 LncRNA SPRY4‐IT1 regulates EZH2 and VEGFA expression in glioma cells

**FIGURE 5:**
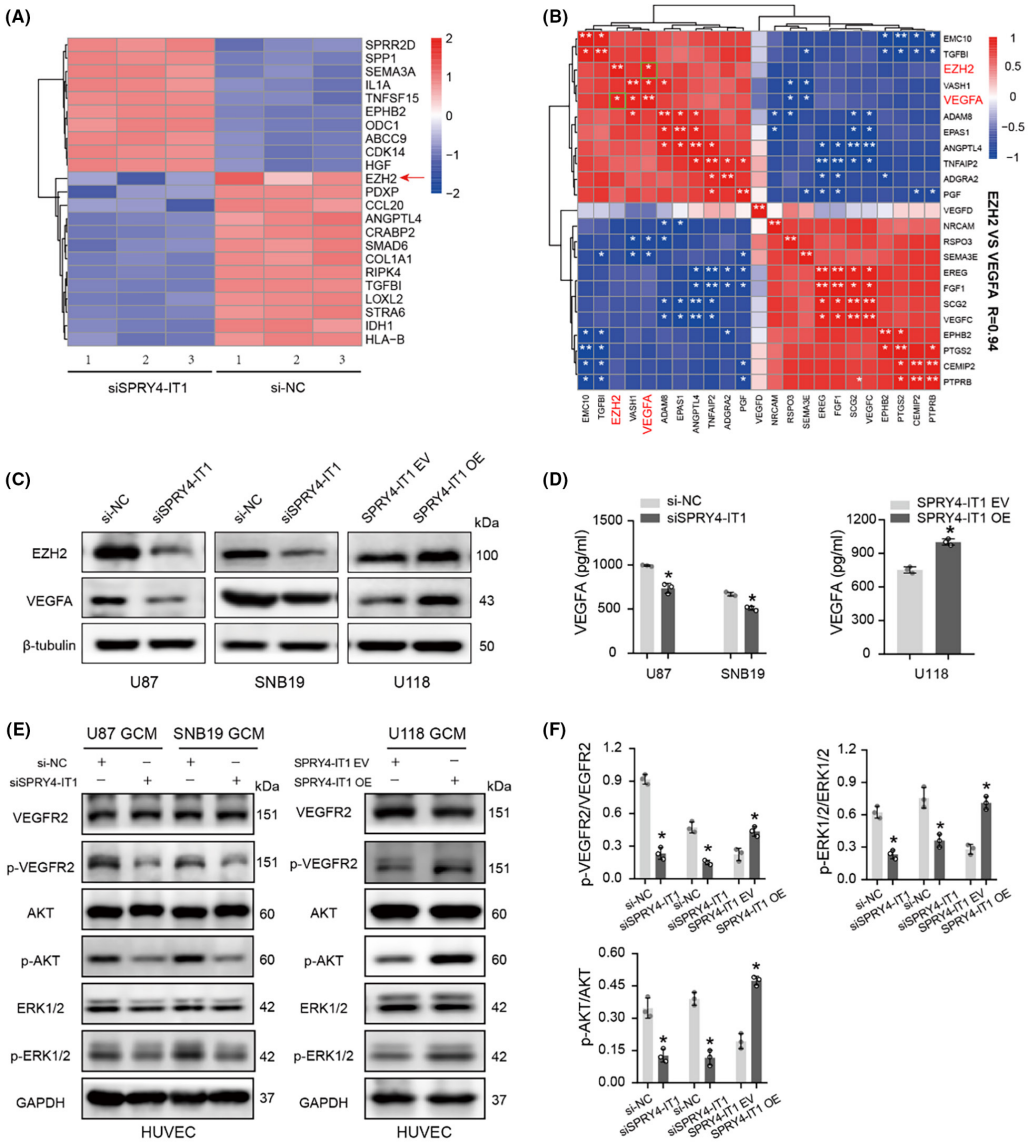
LncRNA SPRY4‐IT1 promotes EZH2 and VEGFA expression in glioma cells and activates VEGFA‐mediated VEGFR2/AKT/ERK1/2 pathway in HUVECs. (A) By silencing SPRY4‐IT1 in U87 cells using siRNA transfection, the differentially expressed genes were identified by RNA‐sequencing and are displayed in the heatmap. The red arrow denotes EZH2. (B) The correlation heatmap displays the correlation between EZH2 and the differentially expressed genes under the angiogenesis GO item. (C) EZH2 and VEGFA protein levels were analyzed by immunoblot in SPRY4‐IT1 knockdown cells and SPRY4‐IT1 overexpression cells. (D) Levels of VEGFA in the CM from glioma cells with SPRY4‐IT1‐silenced or SPRY4‐IT1‐overexpressed were examined by ELISA. (E) The protein expression of VEGFR2, p‐VEGFR2, AKT, p‐AKT, ERK1/2 and p‐ERK1/2 in HUVECs treated with CM from glioma cells with SPRY4‐IT1‐silenced or SPRY4‐IT1‐overexpressed using immunoblot. (F) Statistical analysis of listed proteins relative gray value. *p < 0.05.

We further analyzed data of RNA‐sequencing and identified 23 genes (upregulated 10 genes; downregulated 13 genes) that had the most significant changes in transcription level in a heatmap (Figure 5A). Among the genes with the most significant changes, we were more interested in EZH2, a well‐known tumor gene that drives the malignant progression of various tumors. Next, we selected all the significant genes under the angiogenesis GO item and analyzed their correlation with EZH2 by Spearman analysis. As shown in Figure 5B, the correlation heatmap displayed that only VEGFA and EZH2 were correlated, and the correlation coefficient reached 0.94. Furthermore, immunoblotting analysis was performed to detect whether the expression of EZH2 and VEGFA are regulated by SPRY4‐IT1 in glioma cells. The data revealed that SPRY4‐IT1 could significantly upregulate EZH2 and VEGFA (Figure 5C). Moreover, ELISA results indicated that VEGFA protein levels were downregulated and upregulated in SPRY4‐IT1‐silenced and ‐overexpressed glioma cells, respectively, compared to the control cells (Figure 5D). These data suggest that SPRY4‐IT1 could promote the cell proliferation and angiogenesis of glioma by inducing the expression of EZH2 and VEGFA.

### 3.6 LncRNA SPRY4‐IT1‐overexpressed glioma conditioned medium activates VEGFA‐mediated VEGFR2/AKT/ERK1/2 pathway in HUVECs

We further explored the downstream mechanisms by which SPRY4‐IT1 regulated VEGFA‐treatment on HUVECs. First, western blotting was used to analyze the protein expression of VEGFR2 on HUVECs treated with CM from SPRY4‐IT1‐silenced and SPRY4‐IT1‐overexpressed glioma cells. We found that VEGFA‐induced p‐VEGFR2 on HUVECs was inhibited when treated with CM of SPRY4‐IT1‐silenced cells. On the other hand, p‐VEGFR2 on HUVECs was activated after treatment with CM of SPRY4‐IT1‐overexpressed cells. However, the HUVECs plasma membrane VEGFR2 level did not change significantly. AKT and ERK1/2 are known downstream molecules of VEGFR2. SPRY4‐IT1‐overexpressed CM treatment upregulated the expression of p‐AKT and p‐ERK1/2 in HUVECs, while the opposite results were observed after treatment with SPRY4‐IT1‐knockdown CM (Figure 5E,F). Therefore, these results indicate that SPRY4‐IT1 could promote angiogenesis in glioma cells by activating the VEGFA‐mediated VEGFR2/AKT/ERK1/2 signaling in HUVECs.

### 3.7 LncRNA SPRY4‐IT1 regulates VEGFA expression through the miR‐101‐3p/EZH2 signaling axis in glioma cells

**FIGURE 6:**
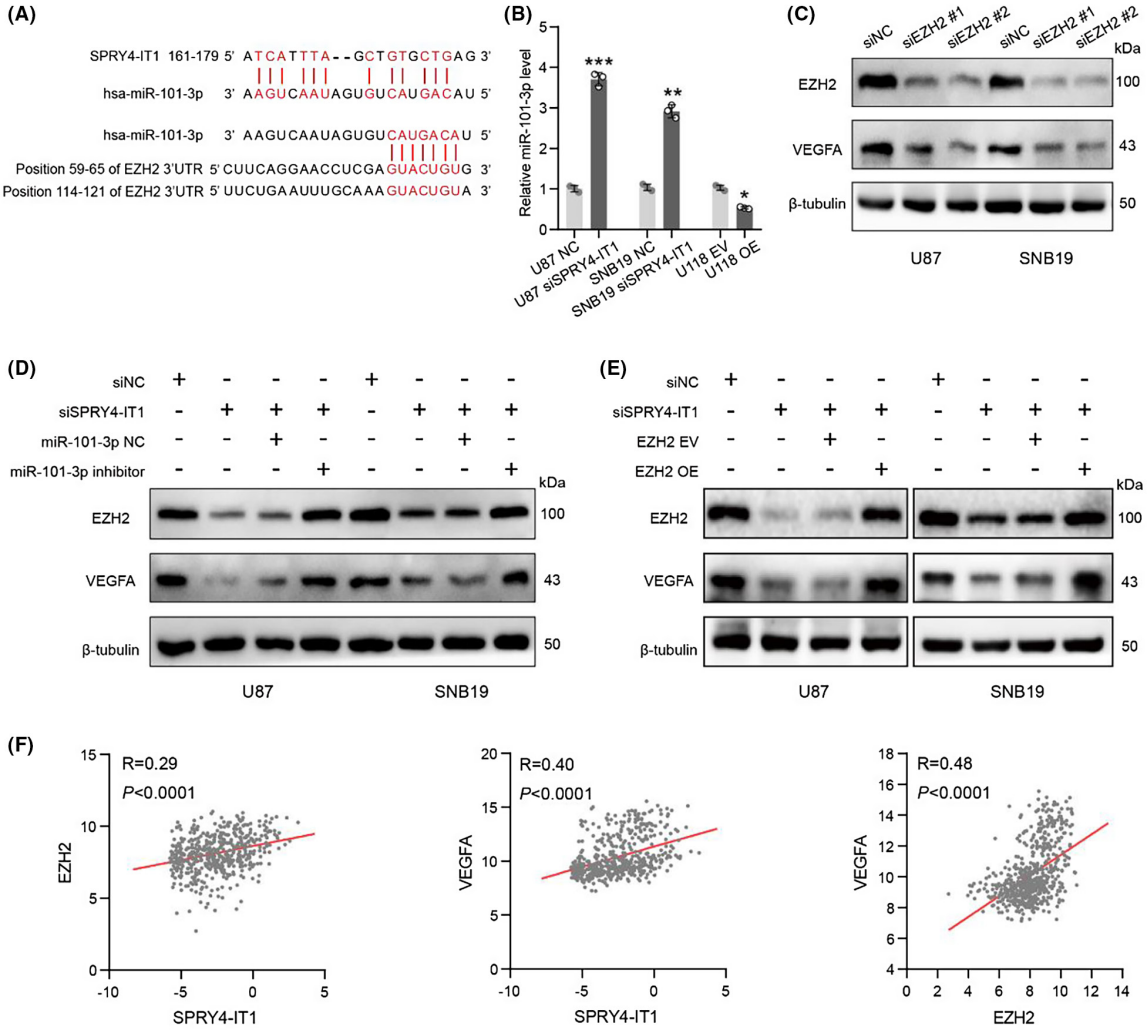
LncRNA SPRY4‐IT1 regulates VEGFA expression in glioma cells via the miR‐101‐3p/EZH2 signaling axis. (A) The predicted binding site between SPRY4‐IT1 and miR‐101‐3p and the putative binding site between miR‐101‐3p and EZH2 3'UTR. (B) miR‐101‐3p expression was detected by qRT‐PCR in glioma cells with SPRY4‐IT1‐silenced or SPRY4‐IT1‐overexpressed. (C) EZH2 and VEGFA protein levels were determined by immunoblot in U87 and SNB19 cells with EZH2‐silenced. (D) EZH2 and VEGFA protein levels were analyzed by immunoblot in U87, SNB19 cells co‐transfected with siSPRY4‐IT1 and miR‐101‐3p inhibitor. (E) EZH2 and VEGFA protein levels were analyzed by immunoblot in U87 and SNB19 cells co‐transfected with siSPRY4‐IT1 and EZH2 overexpression plasmid. (F) The expression correlation between SPRY4‐IT1, EZH2, and VEGFA of patients with glioma from the TCGA dataset. *p < 0.05, **p < 0.01 and ***p < 0.001.

Previous studies reported that lncRNA SPRY4‐IT1 upregulates EZH2 to exert oncogenic properties in cholangiocarcinoma and bladder cancer by sponging miR‐101‐3p.^10^, ^20^ Bioinformatics prediction analysis indicated that SPRY4‐IT1 had putative binding sites with miR‐101‐3p, and miR‐101‐3p had a binding sequence with EZH2 (Figure 6A). Further, we investigated the miR‐101‐3p levels in SPRY4‐IT1‐knockdown and SPRY4‐IT1‐overexpressed glioma cells. qRT‐PCR results demonstrated that miR‐101‐3p levels were significantly upregulated in SPRY4‐IT1‐silenced U87 and SNB19 cells compared with control cells, while overexpression of SPRY4‐IT1 decreased miR‐101‐3p expression in U118 cells (Figure 6B). RNA‐sequencing and western blotting confirmed that SPRY4‐IT1 regulated EZH2 and VEGFA expression in glioma cells. To demonstrate that SPRY4‐IT1 sponges miR‐101‐3p to promote the expression of EZH2 and VEGFA and that EZH2 is upstream of VEGFA, we performed an immunoblot to detect the expression of VEGFA in U87 and SNB19 cells after EZH2 knockdown. The results revealed that VEGFA expression was downregulated following EZH2 silencing (Figure 6C). Next, we co‐transfected siSPRY4‐IT1 and miR‐101‐3p inhibitor in SNB19 and U87 cells, and immunoblot data revealed re‐upregulation of EZH2 and VEGFA protein levels (Figure 6D). Additionally, our immunoblot analysis demonstrated that the EZH2 and VEGFA expression were largely restored in SNB19 and U87 cells co‐transfected with siSPRY4‐IT1 and EZH2 overexpression plasmid compared with SPRY4‐IT1 silenced cells (Figure 6E). We also investigated the correlation between SPRY4‐IT1, EZH2, and VEGFA using the TCGA dataset and found a close expression correlation among SPRY4‐IT1, EZH2, and VEGFA (Figure 6F). Collectively, the data indicate that SPRY4‐IT1 could enhance VEGFA expression via the miR‐101‐3p/EZH2 signaling axis in glioma cells.

### 3.8 LncRNA SPRY4‐IT1 promotes glioma cell proliferation and angiogenesis via the miR‐101‐3p/EZH2/VEGFA signaling axis

**FIGURE 7:**
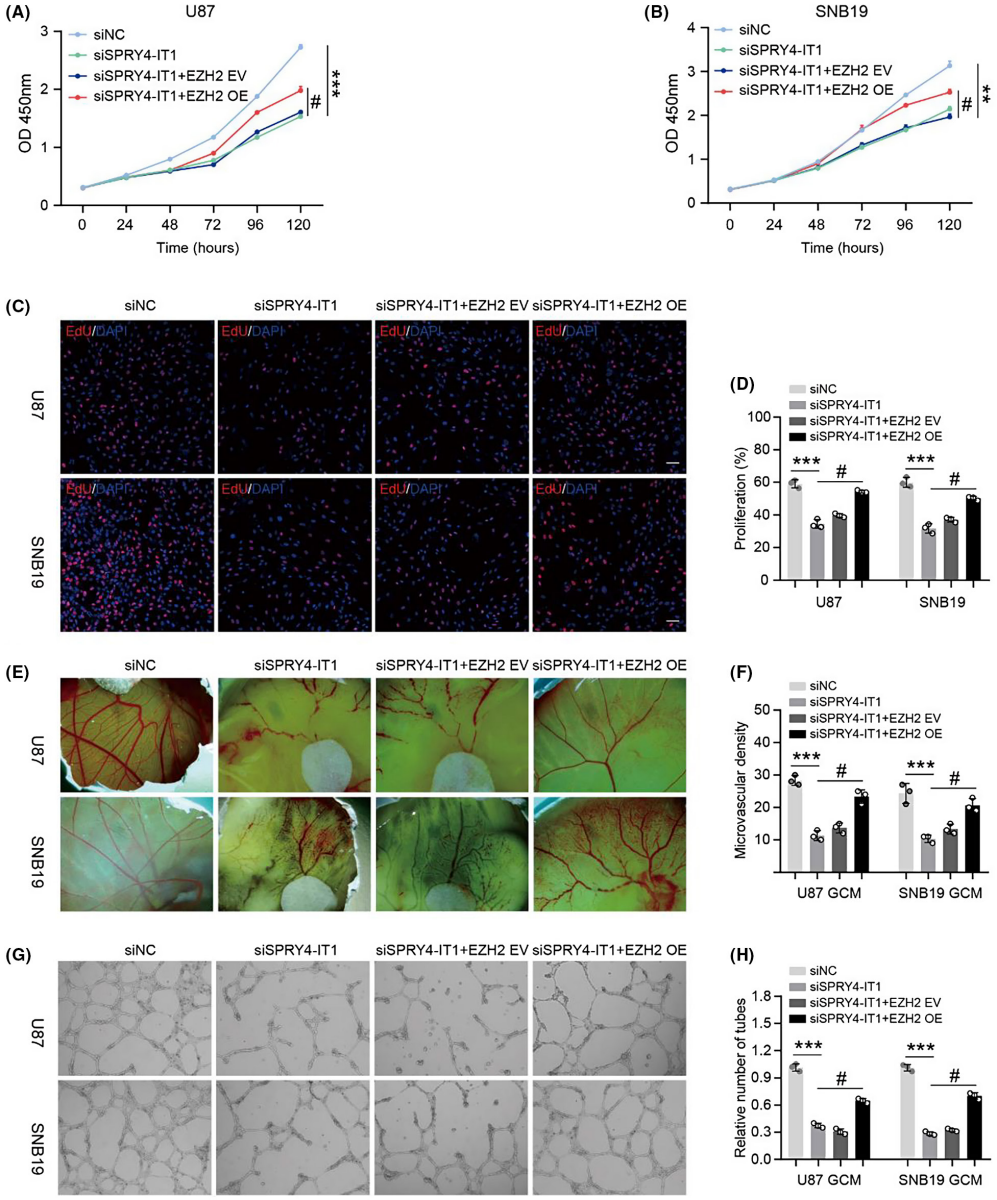
LncRNA SPRY4‐IT1 promotes glioma cell proliferation and angiogenesis by miR‐101‐3p/EZH2 signaling. (A, B) The ability of cell proliferation was examined by CCK‐8 assays in U87 and SNB19 cells co‐transfected with siSPRY4‐IT1 and EZH2 overexpression plasmid. (C) The cell proliferation rate was assessed by EdU assays in U87 and SNB19 cells co‐transfected with siSPRY4‐IT1 and EZH2 overexpression plasmid. (D) Statistical analysis of cell proliferation rate. (E) CAM assays were performed to investigate the effect of CM from U87, SNB19 cells co‐transfected with siSPRY4‐IT1 and EZH2 overexpression plasmid on angiogenesis in vivo. (F) Statistical analysis of angiogenesis. (G) Matrigel tube formation assays were used to measure the effect of CM from U87 and SNB19 cells co‐transfected with siSPRY4‐IT1 and EZH2 overexpression plasmid on tube formation of HUVECs in vitro. (H) Statistical analysis of HUVECs tube formation. Scale bar: 100 μm. #*p* < 0.05, **p < 0.01 and ***p < 0.001.

**FIGURE 8:**
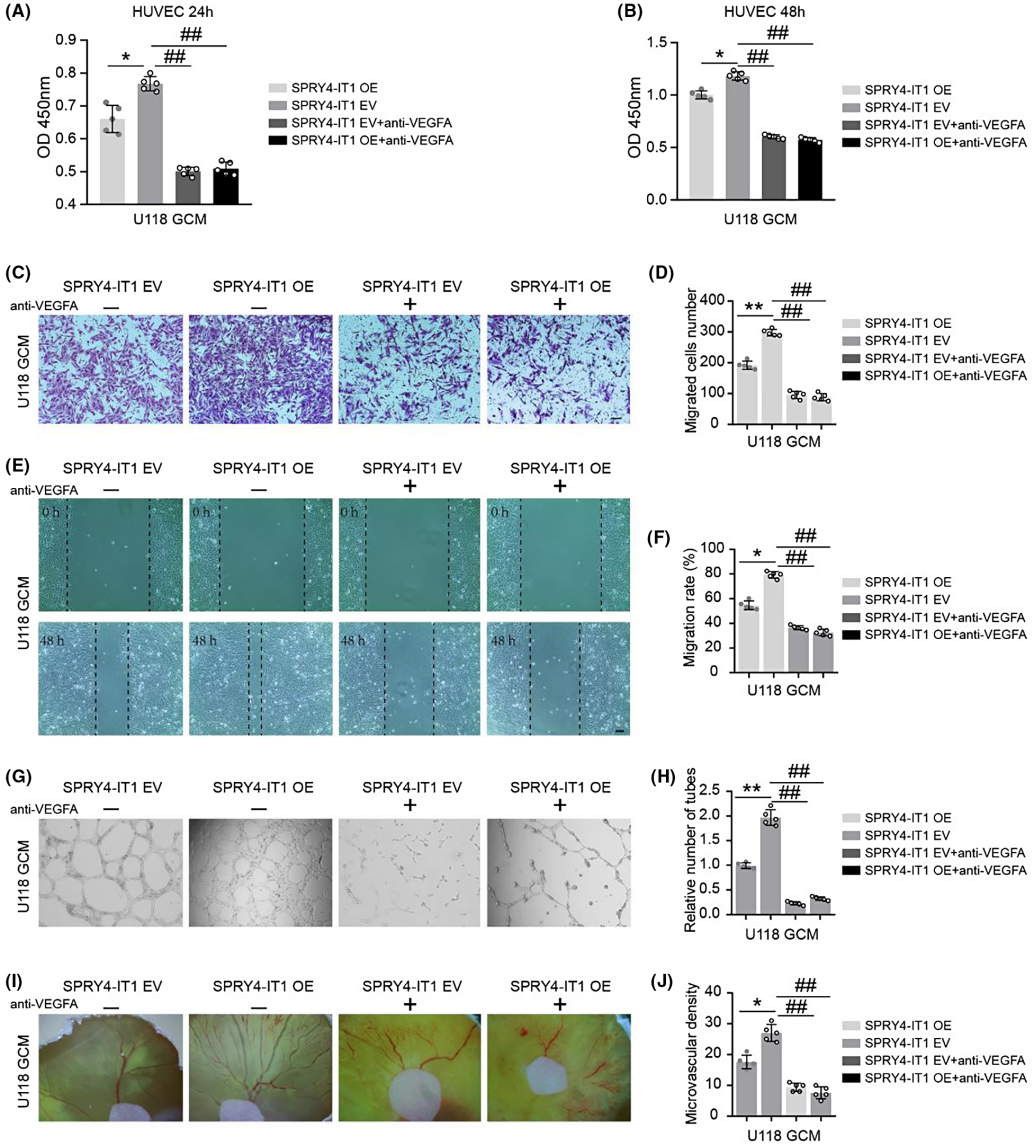
LncRNA SPRY4‐IT1 promotes the ability of glioma cells to induce proliferation, migration, and angiogenesis of HUVECs by VEGFA. (A, B) Cell growth was evaluated using CCK‐8 assays in HUVECs combining SPRY4‐IT1‐overexpressed U118‐GCM and VEGFA‐neutralizing antibody (100 ng/mL) treatment at 24 h and 48 h. (C) Effects of combining SPRY4‐IT1‐overexpressed U118‐GCM and VEGFA‐neutralizing antibody treatment on the migration of HUVECs were tested using transwell assays. Scale bar: 100 μm. (D) Quantitative analysis of migrated HUVECs number. (E) Wound‐healing assays were used to assess the migration ability of HUVECs cultured in combination with SPRY4‐IT1‐overexpressed U118‐GCM and VEGFA‐neutralizing antibody treatment at 48 h. Scale bar: 200 μm. (F) Statistical analysis of HUVECs migration rate. (G) Effects of combining SPRY4‐IT1‐overexpressed U118‐GCM and VEGFA‐neutralizing antibody treatment on tube formation of HUVECs were measured by Matrigel tube formation assay in vitro. (H) Statistical analysis of HUVECs tube formation. (I) The CAM assays were conducted to evaluate the effect of SPRY4‐IT1‐overexpressed U118‐GCM combined with VEGFA‐neutralizing antibody treatment on angiogenesis in vivo. (J) Statistical analysis of angiogenesis. ##*p* < 0.01, *p < 0.05, **p < 0.01.

Here, we performed rescue experiments to investigate whether the miR‐101‐3p/EZH2/VEGFA axis, involved in the lncRNA SPRY4‐IT1, promoted glioma cells proliferation and induced angiogenesis. siSPRY4‐IT1 and EZH2 overexpression plasmid were co‐transfected into U87 and SNB19 cells, then CCK‐8 and EdU tests were performed. The results showed that this co‐transfection partially reversed the proliferation of glioma cells compared with siSPRY4‐IT1 cells (Figure 7A–D). CAM and HUVECs tube formation assays showed that this co‐transfection largely rescued the ability of glioma cells to induce angiogenesis (Figure 7E–H). Next, a VEGFA‐neutralizing antibody was combined with the GCM obtained from SPRY4‐IT1‐overexpressed U118 cells. CCK‐8 results showed that compared with SPRY4‐IT1‐overexpressed U118 GCM treatment alone, the cell viability of HUVECs was decreased after treatment with the VEGFA‐neutralizing antibody (Figure 8A,B). Similar results were observed on transwell and scratch wound assays, which showed that the cell migration capability of HUVECs was decreased when combined with VEGFA‐neutralizing antibody treatment compared with SPRY4‐IT1‐overexpressed U118 GCM treatment alone (Figure 8C–F **)**. HUVECs tube formation and CAM assays demonstrated that the angiogenesis effects induced by SPRY4‐IT1‐overexpressed U118 GCM were weakened when combined with VEGFA‐neutralizing antibody treatment (Figure 8G–J **)**. Therefore, the rescue experiment results demonstrated that the effect of SPRY4‐IT1 on glioma cell proliferation and angiogenesis occurred partly through the miR‐101‐3p/EZH2/VEGFA axis.

### 3.9 LncRNA SPRY4‐IT1 function confirmed in orthotopic nude mice model

**FIGURE 9:**
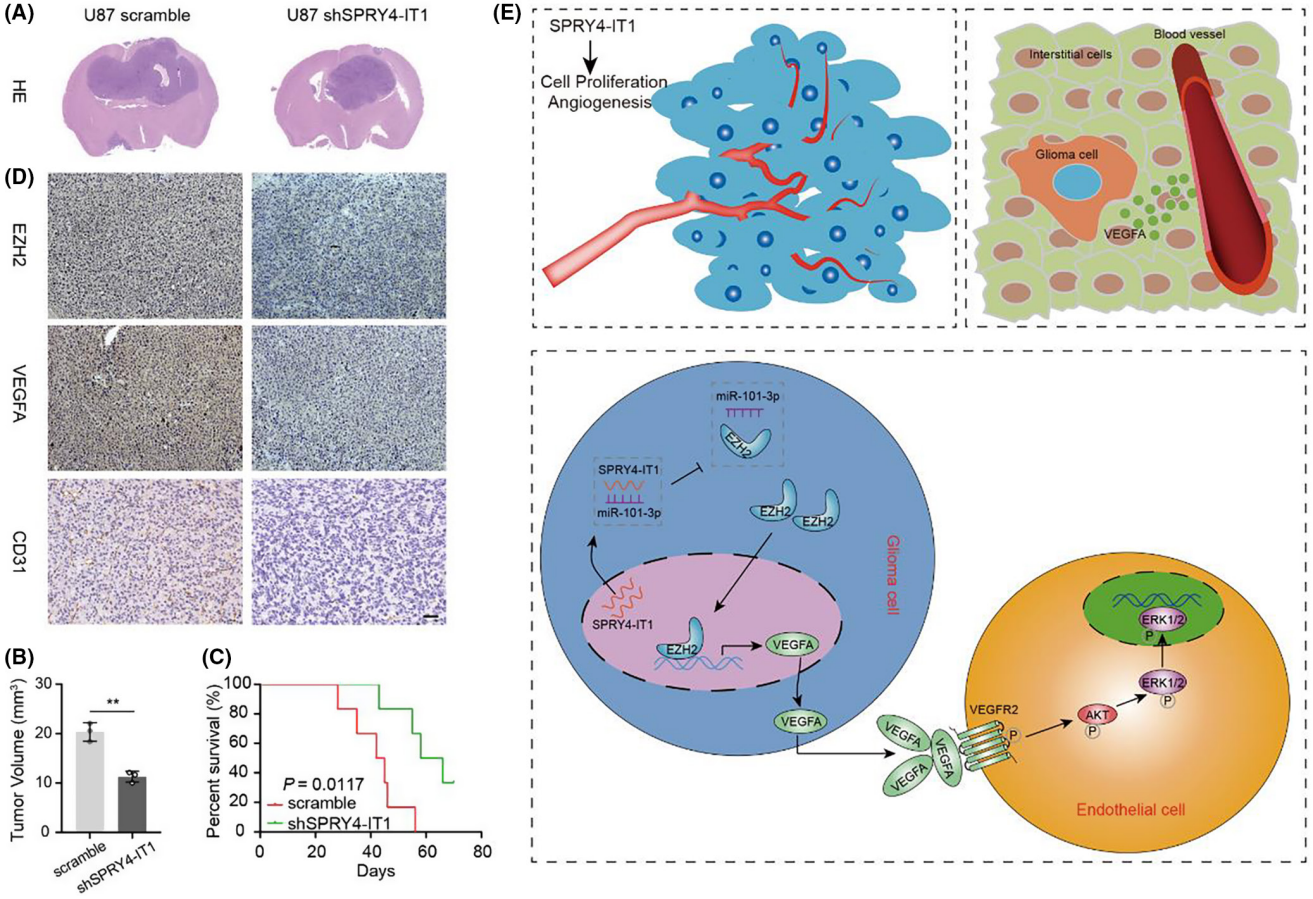
The SPRY4‐IT1/miR‐101‐3p/EZH2/VEGFA signaling axis promotes glioma tumorigenesis and angiogenesis. (A) HE representative images display the size of intracranial tumors of U87 scramble and shSPRY4‐IT1 xenograft mice. (B) Statistical analysis of the size of intracranial tumors. (C) Kaplan–Meier survival curve shows the survival times of U87 scramble and shSPRY4‐IT1 xenograft mice. (D) Immunohistochemical staining representative images show EZH2, VEGFA, and CD31 expression in U87 scramble and shSPRY4‐IT1 xenograft mice. Scale bar: 50 μm. (E) A schematic diagram showing the SPRY4‐IT1/miR‐101‐3p/EZH2/VEGFA signaling axis promoting cell proliferation and angiogenesis. **p < 0.01.

To investigate the relevance of lncRNA SPRY4‐IT1 loss in glioma cells in vivo, we established an U87‐shSPRY4‐IT1 stable cell line. U87‐shSPRY4‐IT1 cells and U87‐scramble cells were orthotopically xenografted by intracerebral injection into immunocompromised mice. As expected, HE staining demonstrated that the tumor size of the U87‐shSPRY4‐IT1 mice was decreased compared to the scramble mice (Figure 9A,B). Moreover, KM survival analysis demonstrated that the survival time of the mice was noticeably prolonged after silencing SPRY4‐IT1 (Figure 9C). Further, immunohistochemistry demonstrated that the expression of EZH2, VEGFA, and CD31 in the in situ tumors of shSPRY4‐IT1 mice were significantly reduced (Figure 9D). The above experiments confirmed that lncRNA SPRY4‐IT1 promoted glioma growth and induced glioma angiogenesis in vivo. A schematic diagram displaying the SPRY4‐IT1/miR‐101‐3p/EZH2/VEGFA signaling axis promoting glioma tumorigenesis and angiogenesis is presented in Figure 9E.

## 4 DISCUSSION

In this study, we provided clinical evidence that lncRNA SPRY4‐IT1 was highly expressed in glioma tissues and was associated with poor patient outcomes. We further elucidated that SPRY4‐IT1 promotes glioma cell proliferation and angiogenesis in vitro and in vivo, and activates the miR‐101‐3p/EZH2/VEGFA pathway. These findings indicate that SPRY4‐IT1 induced glioma tumorigenesis and angiogenesis, and could be a promising prognostic indicator in patients with glioma.

LncRNAs have been regarded as important mediators in glioma development.^21^ Here, we investigated the possible involvement of lncRNA SPRY4‐IT1 in glioma progression. Although previous reports have also identified that SPRY4‐IT1 was upregulated in glioma specimens and was related with poor outcomes in patients with glioma,^14^ the underlying mechanisms of SPRY4‐IT1 in glioma tumorigenesis have not been fully elucidated. In this study, we first demonstrated that SPRY4‐IT1 expression was elevated in glioma and correlated with poor patient prognosis from analysis of the TCGA database and our own specimens. These data are consistent with previous studies and indicate that SPRY4‐IT1 may play an oncogenic role and could be a biomarker in glioma. He et al. and Liu et al. reported that knockdown of SPRY4‐IT1 suppressed U251 and SF295 cell proliferation.^15^, ^22^ In this current study, U87 and SNB19 cells with relatively high SPRY4‐IT1 expression and U118 cells with relatively low SPRY4‐IT1 expression were chosen for our research. We found that SPRY4‐IT1 promoted glioma cell proliferation in a series of functional experiments both in vitro and in vivo.

To further elucidate the molecular mechanism of SPRY4‐IT1 promoting the growth of glioma cells, we performed RNA‐sequencing to compare the transcriptomes of U87 siSPRY4‐IT1‐NC and U87 siSPRY4‐IT1 cells. GO enrichment analysis results revealed that the term of angiogenesis was significantly enriched. Inducing angiogenesis is one of the most crucial hallmarks of cancer, and anti‐angiogenic therapy has recently become one of the key strategies in anti‐tumor therapy.^23^ Angiogenesis is a universal characteristic of glioma progression that facilitates proliferation and migration.^24^ Bevacizumab, a humanized monoclonal antibody against VEGF, was approved as a clinical first‐line chemotherapeutic drug to treat recurrent glioblastoma.^25^ Therefore, anti‐angiogenesis therapy is essential to limit the progression of tumors, including gliomas. Current evidence suggests that lncRNA could exert a modulatory role in glioma angiogenesis, such as H19, XIST, HULC, and PAXIP1‐AS1.^26^, ^27^, ^28^, ^29^ However, the relationship between SPRY4‐IT1 and glioma angiogenesis remained unknown. In this study, we observed that a conditioned medium of glioma cells with knockdown or overexpressed SPRY4‐IT1 could suppress or stimulate the tube formation of HUVECs in vitro and angiogenesis of CAM in vivo, respectively. Our study provides evidence that SPRY4‐IT1 functions as an angiogenesis regulator in glioma. Besides, the clinical glioma specimen's immunohistochemistry results also demonstrated a positive correlation between SPRY4‐IT1 value and vascular endothelial marker (CD34).

Enhancer of zeste homolog 2 (EZH2) is a member of the Polycomb‐group (PcG) family and acts as an important epigenetic transcription regulator. Studies have confirmed EZH2 as an oncogene that could mediate glioma tumorigenesis and angiogenesis.^30^, ^31^, ^32^ It has been reported that SPRY4‐IT1 induces tumor cell growth and metastasis by regulating EZH2.^10^, ^20^, ^33^ Consistently, we found that SPRY4‐IT1 enhanced EZH2 mRNA and protein levels in glioma cells. Previous studies demonstrated EZH2 as a direct target of miR‐101‐3p,^10^, ^34^ and lncRNA SPRY4‐IT1 exerted a ceRNA mechanism by sponging miR‐101‐3p.^10^, ^19^, ^20^, ^35^ In this study, we showed that SPRY4‐IT1 could suppress miR‐101‐3p levels in glioma cells, and EZH2 protein expression was restored in glioma cells after co‐transfection with siSPRY4‐IT1 and miR‐101‐3p inhibitor. Moreover, restoring EZH2 in SPRY4‐IT1‐silenced glioma cells reactivated cell proliferation and angiogenesis. Taken together, these results indicate that SPRY4‐IT1 sponges miR‐101‐3p to induce EZH2‐mediated cell proliferation and angiogenesis in glioma cells.

To further investigate the downstream mechanism of EZH2, we analyzed the correlation between EZH2 and differential genes under the angiogenesis GO item using the Spearman analysis. The results showed that only VEGFA and EZH2 were relevant. VEGFA, a crucial molecular of tumor angiogenesis, plays a critical role in endothelial cell proliferation, migration, and tube formation,^36^ and has also been reported to promote cell cycle, proliferation, and metastasis in glioma as an oncogene.^37^ In our study, we found that the expression of VEGFA was stably downregulated in SPRY4‐IT1‐silenced glioma cells, while VEGFA was strongly upregulated in SPRY4‐IT1‐overexpressed cells. Consistently, the VEGFA concentration in the conditioned medium from SPRY4‐IT1‐silenced or SPRY4‐IT1‐overexpressed glioma cells was decreased and increased, respectively. Liu et al. reported that EZH2 contributed to the progression of lung adenocarcinoma by elevating the expression of VEGFA.^38^ Geng et al. also reported that EZH2 could promote non‐small cell lung cancer progression by upregulating the VEGFA/AKT signaling axis.^39^ Our data demonstrated that VEGFA expression was significantly downregulated following EZH2 inhibition, and VEGFA protein levels were restored in glioma cells after co‐transfection with siSPRY4‐IT1 and EZH2‐overexpression plasmid, suggesting that SPRY4‐IT1 regulated VEGFA expression through the mediation of EZH2 in glioma cells. VEGFA, an effective angiogenic factor, binds to VEGFR2 and activates downstream effect pathways to promote the proliferation and migration of endothelial cells. Sun et al. reported that VEGF could induce VEGFR2 endocytosis and downstream AKT/ERK signaling activation in HUVECs.^40^ Consistent with Sun's report, our results showed that phospho‐VEGFR2, phospho‐AKT, and phospho‐ERK1/2 expression levels were downregulated in HUVECs treatment in a conditioned medium from SPRY4‐IT1‐silenced glioma cells, which significantly weakened HUVECs proliferation and migration viability. Collectively, these data indicated that SPRY4‐IT1 promoted glioma cell proliferation and angiogenesis via the miR‐101‐3p/EZH2 axis by mediating VEGFA downregulation.

In the current clinical treatments of glioma patients, bevacizumab (anti‐VEGF monoclonal antibody) is used as a first‐line chemotherapeutic drug for anti‐angiogenic therapy. Many clinical trials suggested that the clinical prognosis of glioma patients was improved with combined treatment using Temozolomide (TMZ) and bevacizumab.^41^, ^42^ The efficacy of combining lentivirus‐shSPRY4‐IT1 and TMZ (or bevacizumab, or TMZ and bevacizumab) in inhibiting glioma cells or xenograft glioma model should also be investigated. More research is still required to fully elucidate the significance and potential targeting of SPRY4‐IT1.

In summary, the present studies indicate that lncRNA SPRY4‐IT1 regulates VEGFA expression to facilitate cell proliferation and angiogenesis of glioma via the miR‐101‐3p/EZH2 axis, and our findings provide mechanistic insights into potential strategies for anti‐angiogenic therapy in glioma.

## AUTHOR CONTRIBUTIONS

Ji Wang, Yanming Chen, Qing Wang, Hui Xu, Qianqian Jiang, and Man Wang performed the experiments. Chunwang Wu, Pei Yu, Tan Zhang, Zongyu Xiao, and Wenjin Chen analyzed the data. Ji Wang wrote the first draft of the article. Qing Lan conceptualized the project, supervised the experiments, and revised the manuscript.

## CONFLICT OF INTEREST

The authors declare that they have no conflict of interest.

## ETHICS STATEMENT

The use of human tissues was approved by the ethics committee of the Second Affiliated Hospital of Soochow University. All animal studies were approved by the Institutional Animal Care and Use Committee of Soochow University.

## Supporting information


Table S1
Table S2

## Data Availability

All data generated or analyzed during this study are included in this article.
