# Supplementary material for: LncRNA SPRY4‐IT1 facilitates cell proliferation and angiogenesis of glioma via the miR‐101‐3p/EZH2/VEGFA signaling axis
Source: Cancer Med. 2022 Dec 7;12(6):7309–26. doi: 10.1002/cam4.5517 (PMC10067065; doi:10.1002/cam4.5517)
Supplement: Supplementary file 1 — Table S1 Table S2 [file CAM4-12-7309-s001.docx]

**Supplymentary Table 1** The relationship between SPRY4-IT1 expression and the clinicopathological characteristics of glioma patients.

| **Clinicopathological parameter** | **Number** | **SPRY4-IT1** | | ***P* value** |
| --- | --- | --- | --- | --- |
|  |  | **Low cases** | **High cases** |  |
| **Total** | 59 | 30 | 29 |  |
| **Age (year)** |  |  |  | 0.1502 |
| ≥60 | 23 | 9 | 14 |  |
| ＜60 | 36 | 21 | 15 |  |
| **Gender** |  |  |  | 0.5188 |
| Male | 31 | 17 | 14 |  |
| Female | 28 | 13 | 15 |  |
| **WHO grade** |  |  |  | 0.0200* |
| II | 15 | 10 | 5 |  |
| III | 21 | 11 | 10 |  |
| IV | 23 | 9 | 14 |  |
| **CD34 IHC** |  |  |  | 0.0218 * |
| Positive | 20 | 6 | 14 |  |
| Negative | 39 | 24 | 15 |  |

**Supplymentary Table 2** Primer sequences for qRT-PCR.

| **Genes** | **Primer sequence (5'-3')** |
| --- | --- |
| SPRY4-IT1 | Forward: AGCCACATAAATTCAGCAGA |
|  | Reverse: CGATGTAGTAGGATTCCTTTCA |
|  |  |
| miR-101-3p | Forward: GCCGAGTACAGTACTGTGA |
|  | Reverse: CTCAACTGGTGTCGTGGA |
|  | RT stem loop: GTCGTATCCAGTGCAGGGTCCGAGGTATTCGCACTGGATACGACTTCAGT |
| U6 | Forward: GCTTCGGCAGCACATATACTAAAAT |
|  | Reverse: CGCTTCACGAATTTGCGTGTCAT |
|  | RT stem loop: CGCTTCACGAATTTGCGTGTCA |
|  |  |
| GAPDH | Forward: GGGAGCCAAAAGGGTCAT |
|  | Reverse: GAGTCCTTCCACGATACCAA |
